# Supplementary material for: A Likelihood Approach for Real-Time Calibration of Stochastic Compartmental Epidemic Models
Source: PLoS Comput Biol. 2017 Jan 17;13(1):e1005257. doi: 10.1371/journal.pcbi.1005257 (PMC5240920; doi:10.1371/journal.pcbi.1005257)
Supplement: S1 File — (TAR.GZ) [file pcbi.1005257.s014.tar.gz › HSPH_Online-SI-Revision/EnKF07/n10-extreme/n10-extreme_EnKF_table-4.pdf]

1	R0	{2.68692, 12.5884}	{2.12221, 4.30879}	{{0.867621, 2.68006}, {3., 5.14641}}	{32.6689, 66.1931}	inf	652.	1442.32	{358.753, 3272.11}	135.392	0.285127	newly	547.	1509.98	{439., 4010.}	184.83	0.334212	Reff	2.38787	0.361917	{0.108536, 0.615032}	84.8618	ar	9089.	15 680.2	{8213., 21 481.}	80.4732
2	R0	{1.79802, 12.5412}	{2.05068, 9.66218}	{{0.431767, 5.71731}, {3., 14.4287}}	{60.8612, 35.8082}	inf	658.	419.688	{37.5061, 996.982}	80.9788	0.598903	newly	408.	308.53	{15., 829.}	83.424	0.61701	Reff	1.51609	0.403617	{0.0823293, 0.666269}	73.3778	ar	7412.	17 954.3	{16 850., 21 535.}	142.233
3	R0	{2.6174, 10.1105}	{2.53251, 7.14222}	{{2.14099, 3.87718}, {3., 14.6708}}	{16.6915, 42.2812}	inf	487.	2364.71	{652.978, 5398.6}	392.957	0.610142	newly	540.	2792.47	{611., 7254.}	426.254	0.576699	Reff	2.41377	1.01639	{0.32592, 2.19727}	57.8921	ar	9109.	15 592.8	{10 226., 22 805.}	75.9653
4	R0	{2.27532, 14.3149}	{2.21593, 9.50684}	{{0.396676, 5.64722}, {3., 13.9808}}	{41.2434, 33.6099}	inf	1121.	377.46	{49.2048, 295.383}	82.047	0.777462	newly	626.	283.79	{22., 288.}	84.9952	0.749159	Reff	1.73038	0.40905	{0.0827499, 0.664782}	76.3607	ar	8478.	17 744.5	{16 830., 18 232.}	109.3
5	R0	{2.96649, 18.0674}	{1.89426, 4.55595}	{{0.103791, 0.863412}, {3., 5.97143}}	{37.2291, 74.7836}	inf	1477.	597.565	{118.45, 1327.4}	69.4164	0.595651	newly	689.	626.24	{114., 1271.}	71.5385	0.40004	Reff	2.25186	0.32138	{0.00649659, 0.674662}	85.7283	ar	9476.	17 019.8	{15 691., 22 182.}	82.4213
6	R0	{2.94778, 16.0002}	{2.13013, 4.51466}	{{0.977116, 2.68115}, {3., 5.14049}}	{29.3677, 72.2774}	inf	820.	1482.33	{390.402, 4456.65}	100.954	0.233426	newly	489.	1629.37	{367., 5086.}	243.72	0.396826	Reff	2.5348	0.416931	{0.0894124, 1.0405}	83.5517	ar	9321.	14 677.8	{6685., 20 464.}	72.1266
7	R0	{2.17562, 11.7184}	{2.36923, 5.18483}	{{1.72967, 0.779571}, {3., 12.6495}}	{25.6928, 59.914}	inf	569.	2373.7	{499.076, 5523.17}	329.495	0.558306	newly	469.	2763.18	{353., 7496.}	502.588	0.664661	Reff	1.95066	0.612529	{0.209954, 1.33105}	68.8217	ar	8345.	15 426.9	{7823., 24 697.}	90.6081
8	R0	{2.7904, 17.8966}	{2.52011, 5.21788}	{{2.01854, 4.05016}, {3., 7.46766}}	{18.3981, 70.8443}	inf	1183.	250.607	{20.9029, 290.201}	88.0642	1.07265	newly	589.	349.05	{13., 530.}	94.8642	0.816695	Reff	2.25715	0.569684	{0.167069, 1.45113}	75.4189	ar	9285.	17 981.	{16 493., 24 580.}	96.623
9	R0	{2.51574, 16.376}	{2.50062, 5.32227}	{{2.01307, 4.16119}, {3., 7.62618}}	{21.775, 67.4996}	inf	1086.	248.945	{110.74, 348.762}	85.1538	0.890535	newly	556.	325.17	{70., 509.}	89.3903	0.630718	Reff	2.01712	0.557968	{0.171381, 1.45294}	73.3126	ar	8937.	18 229.8	{16 561., 22 596.}	103.981
10	R0	{2.56405, 18.1787}	{2.47919, 5.52744}	{{2.01528, 4.17379}, {3., 7.70673}}	{21.3323, 69.5939}	inf	985.	263.845	{49.898, 269.962}	90.1861	0.916466	newly	476.	364.72	{35., 499.}	105.197	0.649625	Reff	2.09996	0.559212	{0.142527, 1.41855}	74.3483	ar	9005.	17 828.6	{16 549., 20 753.}	100.615
11	R0	{2.475, 17.1709}	{2.39257, 9.51898}	{{0.684937, 6.85937}, {3., 12.0827}}	{31.7549, 44.5633}	inf	1114.	842.652	{25.9413, 2183.86}	106.463	0.900809	newly	539.	611.01	{12., 1879.}	127.935	0.825783	Reff	1.97134	0.633796	{0.0988283, 0.961808}	71.0125	ar	8870.	18 168.9	{15 772., 22 539.}	104.835
12	R0	{2.14974, 11.8619}	{2.43593, 6.00246}	{{1.24473, 4.19507}, {3., 7.7925}}	{26.7072, 49.3971}	inf	514.	225.514	{10.9266, 270.216}	86.6662	0.777098	newly	376.	235.54	{7., 484.}	86.3138	0.707893	Reff	1.93176	0.509119	{0.158905, 1.11838}	74.2064	ar	8414.	17 830.1	{16 471., 19 304.}	111.909
13	R0	{2.20385, 14.4767}	{2.60554, 6.70097}	{{1.68073, 3.53516}, {3., 9.39776}}	{30.0607, 53.7121}	inf	1178.	453.347	{71.1515, 618.078}	83.8891	0.734909	newly	681.	514.22	{99., 673.}	98.7753	0.586599	Reff	1.66898	0.603322	{0.205399, 0.908709}	67.8564	ar	8347.	18 237.2	{16 342., 26 025.}	118.487
14	R0	{2.35671, 13.8357}	{1.936, 4.38204}	{{0.102745, 0.875038}, {3., 5.96699}}	{44.6019, 68.3509}	inf	1020.	585.905	{118.303, 1316.62}	67.9411	0.490755	newly	683.	611.22	{104., 1255.}	71.2064	0.404537	Reff	1.89291	0.31355	{0.0106684, 0.657584}	83.4356	ar	8719.	17 042.7	{15 796., 22 329.}	98.0741
15	R0	{1.91368, 10.1082}	{2.20132, 9.89229}	{{0.419771, 5.69559}, {3., 14.2361}}	{54.9342, 41.5177}	inf	746.	368.808	{43.0546, 303.211}	81.0718	0.659596	newly	600.	277.84	{19., 301.}	84.1933	0.781034	Reff	1.56616	0.418186	{0.0866075, 0.659178}	73.4272	ar	7486.	17 863.	{16 860., 20 227.}	138.619
16	R0	{2.48727, 14.882}	{2.06958, 4.21284}	{{0.140359, 0.881008}, {3., 5.93937}}	{37.9671, 71.6917}	inf	818.	595.958	{149.61, 1395.58}	75.57	0.451283	newly	517.	687.33	{123., 1296.}	103.677	0.386007	Reff	2.15174	0.295415	{0.0224033, 0.460361}	86.2709	ar	8894.	16 809.2	{15 159., 19 630.}	90.7324
17	R0	{2.46795, 13.1915}	{2.37362, 5.61136}	{{1.72832, 0.689082}, {3., 13.14}}	{23.285, 58.9885}	inf	685.	2235.02	{507.371, 5524.31}	244.717	0.495953	newly	499.	2557.82	{243., 7022.}	430.036	0.61631	Reff	2.17698	0.651978	{0.201521, 1.33242}	70.0512	ar	8796.	15 459.7	{8263., 24 155.}	80.5605
18	R0	{2.96603, 16.7543}	{1.97875, 4.097}	{{0.130544, 0.852525}, {3., 5.9739}}	{34.3629, 75.5466}	inf	1177.	548.106	{123.95, 1268.15}	68.4043	0.551809	newly	657.	643.25	{105., 1227.}	76.8508	0.393682	Reff	2.40011	0.327913	{0.0183144, 0.472274}	86.3376	ar	9394.	16 797.4	{15 229., 22 285.}	83.1637
19	R0	{2.25216, 9.90465}	{2.53144, 4.64975}	{{1.81747, 0.825927}, {3., 12.8837}}	{26.4776, 63.6782}	inf	358.	2235.26	{506.088, 5487.16}	529.446	0.677026	newly	390.	2721.09	{432., 7415.}	603.023	0.680129	Reff	2.11343	0.604237	{0.217006, 1.12733}	71.4096	ar	8631.	15 021.1	{9297., 24 504.}	76.8873
20	R0	{2.70837, 18.1923}	{2.5155, 5.5955}	{{2.01986, 4.17489}, {3., 7.70006}}	{19.1137, 69.2425}	inf	1099.	269.871	{36.4604, 264.251}	90.5461	0.969366	newly	555.	374.35	{26., 502.}	102.132	0.714145	Reff	2.21924	0.563434	{0.176454, 1.4397}	75.3188	ar	9183.	17 934.5	{16 566., 21 130.}	97.8735
21	R0	{2.30288, 15.5877}	{2.48098, 9.12323}	{{0.953745, 1.57037}, {3., 19.4414}}	{30.3988, 60.6406}	inf	1079.	438.25	{78.0663, 837.391}	84.8171	0.778565	newly	546.	1004.25	{60., 2061.}	205.511	0.710003	Reff	1.82273	0.63579	{0.171055, 0.977065}	67.5982	ar	8674.	18 199.8	{16 493., 21 746.}	109.82
22	R0	{2.82208, 10.7544}	{2.50333, 7.93674}	{{2.12808, 4.0455}, {3., 14.7051}}	{16.7168, 42.8338}	inf	295.	2161.57	{646.161, 3711.09}	640.731	0.797908	newly	339.	2201.01	{538., 6156.}	557.159	0.678923	Reff	2.6838	0.897312	{0.307674, 2.09766}	66.5656	ar	9333.	14 529.1	{7911., 21 957.}	62.625
23	R0	{1.69923, 10.1366}	{2.4398, 5.2889}	{{1.46494, 2.32892}, {3., 9.88334}}	{50.6764, 47.8237}	inf	645.	400.297	{16.3247, 1056.99}	93.4182	0.761893	newly	502.	786.8	{12., 4244.}	170.279	0.770975	Reff	1.4056	0.668803	{0.0433236, 1.45985}	59.3446	ar	7055.	18 658.1	{16 682., 24 982.}	164.466
24	R0	{2.64944, 19.054}	{2.4862, 8.53679}	{{0.905129, 1.50774}, {3., 18.8866}}	{22.506, 55.518}	inf	1801.	387.519	{44.0616, 350.112}	84.3243	1.00653	newly	721.	875.19	{20., 1922.}	152.201	0.815092	Reff	1.75684	0.589	{0.0988639, 0.912842}	69.0826	ar	9147.	18 002.4	{16 531., 21 419.}	96.8122
25	R0	{2.53191, 10.9214}	{2.47523, 5.80916}	{{1.24195, 4.19873}, {3., 7.76299}}	{22.2054, 46.8094}	inf	473.	257.598	{11.6219, 267.701}	97.4213	0.758252	newly	441.	260.94	{8., 392.}	100.122	0.800043	Reff	2.32379	0.395824	{0.156346, 0.619107}	82.9664	ar	9009.	17 458.6	{16 486., 18 949.}	94.1289
26	R0	{1.89155, 6.76944}	{2.38735, 5.72691}	{{1.72297, 0.667157}, {3., 13.1479}}	{31.7049, 65.7222}	inf	395.	2232.51	{517.19, 5523.34}	476.668	0.675527	newly	577.	2537.68	{243., 7116.}	359.858	0.574403	Reff	1.71999	0.656722	{0.203733, 1.33611}	62.8021	ar	7348.	15 536.	{8179., 24 377.}	113.601
27	R0	{2.63149, 15.6979}	{2.10536, 4.24217}	{{0.136197, 0.873282}, {3., 5.95091}}	{35.1179, 72.9762}	inf	797.	566.78	{144.011, 1316.17}	72.2572	0.446788	newly	482.	596.55	{117., 1208.}	86.9357	0.359537	Reff	2.28124	0.289478	{0.0200813, 0.459736}	87.3105	ar	9104.	16 711.1	{15 190., 18 912.}	86.8647
28	R0	{2.85141, 12.6992}	{2.5687, 7.02609}	{{2.14398, 3.84061}, {3., 14.6575}}	{15.0215, 49.3182}	inf	622.	2476.47	{593.803, 5807.34}	309.071	0.529062	newly	530.	2702.78	{562., 7000.}	420.491	0.576439	Reff	2.57824	0.990164	{0.321909, 2.19801}	61.5954	ar	9317.	15 799.	{9038., 22 965.}	75.9331
29	R0	{2.54244, 12.9085}	{2.41309, 5.4054}	{{1.46687, 2.32369}, {3., 9.8964}}	{23.4445, 58.1253}	inf	820.	358.491	{20.6852, 847.014}	87.727	0.815331	newly	612.	672.69	{22., 3155.}	135.087	0.767372	Reff	2.19184	0.619573	{0.0533864, 1.444}	72.4085	ar	9034.	18 380.7	{16 705., 23 372.}	103.462
30	R0	{2.10982, 11.7636}	{2.43648, 5.46916}	{{1.46841, 2.21643}, {3., 9.82836}}	{33.236, 53.5078}	inf	762.	353.847	{18.0278, 1061.17}	83.5438	0.816771	newly	521.	716.89	{14., 3181.}	153.196	0.776755	Reff	1.77373	0.633763	{0.059072, 1.46155}	66.9355	ar	8224.	18 635.3	{16 722., 25 054.}	126.596
31	R0	{2.76312, 15.5396}	{2.19382, 4.39214}	{{0.971197, 2.68207}, {3., 5.15878}}	{28.4907, 71.7358}	inf	1053.	1381.23	{525.759, 2680.64}	65.8786	0.201487	newly	607.	1554.86	{495., 3185.}	167.72	0.320489	Reff	2.27211	0.409669	{0.077802, 0.85446}	81.9697	ar	9220.	15 687.3	{7654., 19 774.}	77.5586
32	R0	{2.21727, 15.5439}	{2.76448, 10.5073}	{{2.317, 3.80922}, {3., 14.4267}}	{24.6794, 32.4027}	inf	1094.	610.335	{116.295, 1140.17}	72.595	0.529202	newly	577.	415.	{59., 623.}	68.617	0.448549	Reff	1.72703	0.63064	{0.219178, 1.15345}	66.4498	ar	8359.	17 586.4	{15 604., 22 883.}	110.388
33	R0	{2.72912, 10.8159}	{2.08642, 4.3074}	{{0.883846, 2.68006}, {3., 5.1232}}	{32.6813, 61.0202}	inf	759.	1330.91	{363.236, 2525.81}	95.0621	0.22509	newly	733.	1389.08	{430., 3335.}	104.385	0.219862	Reff	2.39835	0.363256	{0.0880808, 0.618173}	84.8632	ar	9196.	15 842.7	{8359., 18 977.}	78.9265
34	R0	{2.20928, 10.1308}	{2.55643, 6.41813}	{{2.14363, 3.55777}, {3., 14.6175}}	{19.3433, 47.4032}	inf	601.	2072.73	{673.252, 3481.4}	253.44	0.49829	newly	568.	2645.13	{626., 7251.}	374.66	0.556812	Reff	1.96317	0.985027	{0.301208, 2.20263}	53.3968	ar	8388.	14 923.2	{8503., 22 577.}	82.096
35	R0	{2.10103, 15.2673}	{2.39851, 5.36386}	{{1.45682, 2.13004}, {3., 9.85534}}	{34.0089, 64.867}	inf	1074.	479.51	{25.7792, 1077.75}	85.1016	0.861332	newly	563.	829.41	{28., 4182.}	163.078	0.758458	Reff	1.64364	0.620985	{0.0616137, 1.45747}	65.3738	ar	8264.	19 026.4	{16 841., 25 279.}	130.232
36	R0	{2.74914, 19.2775}	{2.4802, 9.68969}	{{0.973862, 1.72005}, {3., 19.4557}}	{20.5549, 50.2724}	inf	1325.	427.679	{82.0886, 843.94}	82.2441	0.819358	newly	562.	946.66	{62., 1923.}	191.609	0.697848	Reff	2.13223	0.665147	{0.154828, 1.22549}	68.8052	ar	9301.	18 337.	{16 527., 24 424.}	97.1503
37	R0	{2.78161, 16.0136}	{2.50527, 5.33482}	{{2.01927, 4.17571}, {3., 7.63998}}	{18.5709, 66.6857}	inf	1098.	253.78	{106.161, 352.738}	84.6799	0.90658	newly	605.	331.72	{71., 524.}	88.8628	0.675193	Reff	2.28481	0.560777	{0.173391, 1.45519}	76.0821	ar	9240.	18 195.4	{16 499., 23 336.}	96.9194
38	R0	{2.4454, 8.33501}	{2.56432, 5.29898}	{{1.82372, 0.808944}, {3., 13.0898}}	{23.3404, 61.1499}	inf	351.	2309.4	{501.282, 5482.88}	559.826	0.695681	newly	491.	2622.37	{506., 6815.}	439.652	0.57498	Reff	2.30772	0.569636	{0.20342, 1.00295}	75.3161	ar	8853.	14 669.9	{8702., 23 457.}	69.0801
39	R0	{2.9154, 12.3861}	{2.39678, 5.66427}	{{1.78368, 0.708283}, {3., 13.0158}}	{20.5166, 58.0079}	inf	746.	2222.16	{517.37, 5512.89}	216.816	0.463145	newly	645.	2701.75	{286., 8001.}	339.943	0.56272	Reff	2.57605	0.677431	{0.243001, 1.33756}	73.7027	ar	9371.	15 775.6	{9462., 24 862.}	73.0743
40	R0	{2.40923, 15.1963}	{2.47966, 5.37938}	{{2.01131, 4.17674}, {3., 7.66684}}	{23.5597, 64.6007}	inf	1012.	254.284	{63.3356, 355.64}	85.4763	0.893347	newly	547.	331.38	{66., 496.}	92.1645	0.660508	Reff	1.91847	0.56384	{0.164291, 1.43289}	72.4131	ar	8735.	18 204.3	{16 518., 24 062.}	108.407
41	R0	{2.63137, 15.238}	{2.41486, 5.41809}	{{1.46144, 2.28625}, {3., 9.80776}}	{23.358, 64.4436}	inf	1144.	516.544	{21.5344, 1085.14}	90.4555	0.960401	newly	680.	818.19	{14., 4310.}	146.21	0.853369	Reff	2.09194	0.632732	{0.0742866, 1.4621}	71.4413	ar	9150.	18 982.2	{16 715., 25 217.}	107.456
42	R0	{2.48769, 19.4593}	{2.8061, 10.1188}	{{2.30795, 3.68567}, {3., 14.998}}	{16.0319, 48.0002}	inf	1677.	661.553	{165.55, 1501.08}	76.7439	0.648822	newly	673.	486.47	{144., 734.}	77.9747	0.454047	Reff	1.67844	0.562084	{0.233121, 0.878351}	66.8513	ar	8932.	17 197.	{15 624., 19 443.}	92.5329
43	R0	{2.61199, 10.214}	{2.55211, 7.21329}	{{2.13023, 3.86514}, {3., 14.6921}}	{16.5533, 41.7581}	inf	259.	2181.63	{645.886, 4260.45}	747.574	0.836644	newly	315.	2281.5	{600., 5800.}	630.438	0.733574	Reff	2.50594	0.883257	{0.299478, 1.8075}	64.7535	ar	9061.	14 511.2	{8658., 21 262.}	64.3893
44	R0	{2.50994, 18.3382}	{2.24549, 8.20664}	{{0.681796, 0.0071352}, {3., 12.2126}}	{33.9659, 55.2484}	inf	1225.	887.836	{24.107, 1685.1}	106.474	0.891865	newly	597.	857.02	{16., 2558.}	153.015	0.810124	Reff	1.9314	0.551361	{0.0740881, 0.809734}	73.8207	ar	8827.	17 941.1	{15 680., 22 611.}	103.252
45	R0	{2.44283, 16.8338}	{2.40139, 5.37593}	{{1.46276, 2.15374}, {3., 9.88185}}	{25.7837, 68.0647}	inf	1233.	402.018	{21.2123, 1072.99}	81.581	0.938888	newly	606.	723.56	{23., 4221.}	139.746	0.770408	Reff	1.87707	0.609861	{0.0578194, 1.45646}	69.4425	ar	8878.	18 772.8	{16 837., 25 057.}	111.453
46	R0	{2.51815, 18.1462}	{2.47767, 9.64383}	{{0.969035, 1.63542}, {3., 19.4293}}	{24.7906, 51.2283}	inf	1241.	425.84	{78.3947, 859.799}	81.7759	0.798646	newly	572.	942.87	{65., 1961.}	188.778	0.699276	Reff	1.943	0.672295	{0.255354, 1.19124}	67.0323	ar	8978.	18 370.9	{16 554., 24 140.}	104.621
47	R0	{2.88885, 16.8987}	{2.44211, 5.51945}	{{1.47045, 2.38179}, {3., 9.93499}}	{19.5427, 67.338}	inf	890.	391.151	{20.3583, 1046.04}	85.169	0.827611	newly	505.	834.37	{18., 4311.}	177.451	0.758526	Reff	2.47083	0.638194	{0.0409986, 1.47031}	74.2128	ar	9337.	18 625.1	{16 709., 25 082.}	99.476
48	R0	{2.58709, 13.8786}	{2.11797, 4.43147}	{{0.89067, 2.6804}, {3., 5.14609}}	{33.3707, 68.2833}	inf	706.	1468.56	{378.659, 4959.42}	122.624	0.258326	newly	483.	1524.76	{405., 5215.}	223.673	0.379088	Reff	2.27612	0.357676	{0.113683, 0.575842}	84.4036	ar	9146.	15 905.1	{8369., 23 511.}	80.7205
49	R0	{2.42959, 10.7132}	{2.50337, 5.05379}	{{1.70856, 0.746404}, {3., 13.0405}}	{23.6251, 61.6261}	inf	413.	2289.31	{508.098, 5487.12}	460.66	0.628364	newly	380.	2549.45	{490., 6790.}	575.771	0.665959	Reff	2.25199	0.578198	{0.211656, 1.06758}	74.325	ar	8863.	14 966.7	{9352., 24 414.}	72.994
50	R0	{2.60513, 13.2796}	{2.0502, 3.74984}	{{0.144866, 0.872541}, {3., 5.95312}}	{36.7619, 72.2375}	inf	466.	615.497	{136.23, 1398.97}	84.7807	0.292561	newly	376.	685.21	{113., 1329.}	124.737	0.351746	Reff	2.3863	0.30995	{0.0231427, 0.459342}	87.0113	ar	9095.	16 142.9	{13 953., 18 877.}	82.4629
